# Legume germination is delayed in dry soils and in sterile soils devoid of microbial mutualists: Species‐specific implications for upward range expansions

**DOI:** 10.1002/ece3.9186

**Published:** 2022-08-23

**Authors:** Andrea M. Keeler, Nicole E. Rafferty

**Affiliations:** ^1^ Department of Evolution, Ecology, and Organismal Biology University of California, Riverside Riverside California USA; ^2^ Rocky Mountain Biological Laboratory Crested Butte Colorado USA

**Keywords:** climate change, distribution, germination, legume, microbe, mutualism

## Abstract

Climate change is affecting species and their mutualists and can lead to the weakening or loss of important interspecific interactions. Through independent shifts in partner phenology and distribution, climatic stress can separate mutualists temporally or spatially, leading to alterations in partner functional traits and fitness. Here, we explored the effects of the loss of microbial mutualists on legume germination success and phenology. In particular, we assessed the effects of mutualism loss via soil sterilization, increased drought, and introduction to novel soils found beyond the current distributions of two focal legume species in subalpine environments. Through common garden experiments in controlled environments, we found evidence that soil sterilization (and consequent microbial absence) and dry soils caused species‐specific phenological delays of 2–5 weeks in germination, likely as a result of interaction loss between legumes and specialized germination‐promoting soil microbes, such as mutualistic rhizobia. Delays in germination caused by a mismatch between legumes and beneficial microbes could negatively affect legume fitness through increased plant–plant competition later in the season. Additionally, we found evidence of the presence of beneficial microbes beyond the current elevational range of one of our focal legumes, which may allow for expansion of the leading edge, although harsh abiotic factors in the alpine may hinder this. Alterations in the strength of soil microbe‐legume mutualisms may lead to reduced fitness and altered demography for both soil microbes and legumes.

## Graphical abstract

In this article, we assess the effects of mutualism loss via soil sterilization, drought, or introduction to novel soils found beyond the current distributions of two focal legume species in subalpine environments. Through common garden experiments in controlled environments, we find evidence that soil sterilization (and consequent microbial absence) and dry soils cause phenological delays of 2‐5 weeks in germination date, likely as a result of interaction loss between legumes and germination‐promoting soil microbes, such as mutualistic rhizobia.

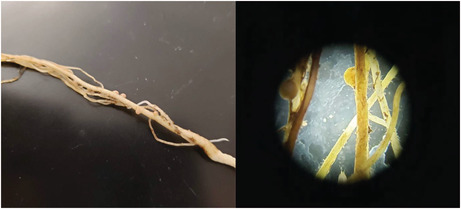

## 1 INTRODUCTION

Mutualisms are essential and beneficial species interactions which profoundly influence the structure, productivity, and stability of communities (Bruno et al., 2003; Leff et al., 2018; Wardle et al., 2004). Mutualistic interactions provide ecosystem services such as nutrient cycling (Wall & Moore, 1999) and increase partner stress tolerance (David et al., 2020; de Zelicourt et al., 2013; Lau & Lennon, 2012). Mutualisms between soil microbes and plants, such as the rhizobia‐legume and the nearly ubiquitous mycorrhizal fungi–plant symbioses, are especially ecologically important for plant growth and fitness, as well as community composition and dynamics (Klironomos, 2002; Mangan et al., 2010; Reynolds et al., 2003; Van Der Heijden & Bardgett, 2008). These mutualisms may become increasingly important as the environment becomes more stressful as a result of anthropogenically induced global climate change (Allan & Soden, 2008; Dai, 2012; Gehring et al., 2017; Lau & Lennon, 2012; Porter et al., 2020). Environmental context can determine the level of investment made by mutualists, such that the net benefits of mutualisms are often greater in more stressful environments (Pringle et al., 2013; Remke et al., 2021). However, some soil microbes enter a state of dormancy in stressful environments, meaning they do not interact with the plant host under increased stress, which can affect host plant distributions and functional traits (Stanton‐Geddes & Anderson, 2011; Simonsen et al., 2017; Werner et al., 2018). Indeed, declines in active partner abundances can destabilize mutualisms (Kiers et al., 2010; Tylianakis et al., 2008), altering plant functional traits and overall fitness (Fitzpatrick et al., 2019; Worchel et al., 2013).

Mutualistic interactions may become decoupled if one partner is dormant or nonreceptive for part of the year while the other is not (Rafferty et al., 2015). Bacteria in particular, including the naturally and agriculturally important nitrogen (N)‐fixing rhizobial bacteria (Harris et al., 1985) and other plant growth‐promoting rhizobacteria (PGPR), are susceptible to desiccation and death in dry conditions (de Vries et al., 2018; de Vries & Shade, 2013; Ngumbi & Kloepper, 2016; Schimel et al., 2007; Xu & Coleman‐Derr, 2019). To avoid losing water to their environment, many bacterial cells can enter a state of dormancy (Lennon & Jones, 2011; Schimel, 2018). In N‐fixing rhizobia, soil drying has been shown to induce dormancy in free‐living cells and inhibit N‐fixation in symbiotic bacteroid cells, leading to denodulation (Aldasoro et al., 2019; Hungria & Vargas, 2000; Vriezen et al., 2006; Zahran, 1999) and short‐term mutualism loss. Similarly, active, free‐living bacterial cells in dry soils may have difficulty forming interactions with plant roots because low soil moisture negatively affects the signaling abilities of soil bacteria and plants (Schimel, 2018; Williams & de Vries, 2020). As a result, the mutualism between legumes and soil microbial species, such as PGPR, can weaken in the short term due to climate change‐induced soil drying.

When active, soil microbial mutualists often ameliorate environmental stress and help host plants overcome limitations, allowing plants to persist in conditions otherwise intolerable (Bennett & Meek, 2020; David et al., 2018; David et al., 2020; Defossez et al., 2011; Lau & Lennon, 2012; Petipas et al., 2017; Redman et al., 2011; Rodríguez‐Echeverría et al., 2016). For this reason, soil microbe–plant mutualistic partners are often able to inhabit a broad range of habitats (Afkhami et al., 2014; Bruno et al., 2003; Harrison et al., 2018; Rodriguez‐Cabal et al., 2012; Stachowicz, 2001). For example, by increasing plant access to N, phosphorus (P), and water, N‐fixing bacteria and arbuscular mycorrhizal fungi (AMF) allow plants to expand their ranges into otherwise unsuitable, nutrient‐poor habitats (Afkhami et al., 2014; Halvorson et al., 1991; Harrison et al., 2018; Hayward et al., 2015; Stachowicz, 2001). Additionally, agricultural studies have demonstrated that some soil microbial species are able to promote host seed germination by excreting phytohormones, thereby increasing germination success in newly colonized habitats (Atzorn et al., 1988; Noel et al., 1996; Bastian et al., 1998; Tsavkelova et al., 2007; Miransari & Smith, 2009; Kumar et al., 2011; Namvar & Sharifi, 2011; Meena et al., 2012; Ngumbi & Kloepper, 2016; Wu et al., 2016). Conversely, the absence of mutualists can negatively affect population persistence and limit species distributions (Benning & Moeller, 2021b; Harrower & Gilbert, 2018; Mueller et al., 2011; Nuñez et al., 2009; Pellmyr, 2003). Indeed, this has been documented in some soil microbe–plant mutualisms (Simonsen et al., 2017; Stanton‐Geddes & Anderson, 2011). Thus, the ability of a plant to successfully establish in a new habitat depends on not only dispersal and the physical conditions in the novel range but also biotic factors, including the presence of mutualists (Brown & Vellend, 2014; HilleRisLambers et al., 2012; van der Putten et al., 2010).

The role of soil microbe–plant mutualisms in shaping the geographic distributions of plant species is still little understood (Benning & Moeller, 2021a; Bueno de Mesquita et al., 2016; Classen et al., 2015). As plants, including legumes, continue to expand their leading range edges up latitudinally and poleward latitudinally in response to climate change (Chen et al., 2011; Harrison et al., 2018), they may encounter harsh environments, made potentially more stressful by the absence of mutualistic partners. Legumes often require exposure to soils that have been pre‐inoculated with compatible soil microbes to establish and persist in dry N‐ and P‐poor soils such as those found in the alpine and subalpine (Bueno de Mesquita et al., 2020; Darcy et al., 2018; Parker, 2001; Simonsen et al., 2017). The absence or reduced abundance of microbial mutualists beyond the current range of a population could impair plant fitness and hinder leading range expansion (Hu et al., 2022; Lankau & Keymer, 2016; Miransari, 2010; Peay et al., 2010; Sedlacek et al., 2014; Wu & Ying‐Ning, 2017). Non‐co‐dispersed, horizontally transmitted symbionts, including legumes, rhizobia, and some other PGPR, may be at high risk of becoming spatially mismatched as they may track climate differently (Keeler et al., 2021). Legumes that interact with specialized mutualists may be less likely to find a compatible partner in novel habitats and thus may fail to establish (Simonsen et al., 2017), while legumes that have been successful in expanding into novel ranges without a historical partner may have benefitted from their ability to relax their partner choice mechanisms and establish interactions with generalist mutualists (Harrison et al., 2017; Younginger & Friesen, 2019). However, compared with the historical interactions, new interactions in a novel habitat may not confer equivalent benefits to the host plant (Bueno de Mesquita et al., 2018; Werner et al., 2018).

A spatial or dormancy‐induced loss of a mutualism, even for part of a season, could lead to reduced germination stimulation by soil microbes which could decrease plant germination success (David et al., 2020; Eldridge et al., 2021), delay host plant germination phenology, and alter downstream phenophases, such as flowering onset (Namvar & Sharifi, 2011), which will alter phenological overlap and interaction strengths among host plants and pollinators (Rafferty & Ives, 2012; Rafferty, Bertelsen and Bronstein, 2016). A shift in flowering phenology without a corresponding shift in pollinator phenology could decrease the fitness of both mutualists (Kudo & Cooper, 2019; Rafferty & Ives, 2011; Schenk et al., 2018). Germination phenology is especially important, as germinants are particularly vulnerable to stress relative to other stages of the plant life cycle. Seedlings that emerge in stressful environments, including in environments lacking certain facultative mutualists, have lower survival (Donohue et al., 2010). Germination timing also shapes competitive outcomes and reproductive success (Fowler, 1984; Leverett, 2017). Studies evaluating the interactions between seeds and soil microbes during the germination process in natural systems are limited (but see: Nelson et al., 2018; Shade et al., 2017; Billingsley Tobias et al., 2017), although the importance of these interactions could become greater in a changing climate; seed germination stimulation by microbes may strengthen (David et al., 2020), or weaken via environmental stress‐induced microbial dormancy (Schimel, 2018).

Here, we explore how germination traits of two legumes are affected by the absence of their mutualisms with soil microbial species. Environmental stress, namely drought, and the possible absence of suitable soil microbes in the expected future ranges of our focal legumes may affect legume germination success and timing. Because germination success can be stimulated by microbes, we hypothesize that legumes in sterilized soils devoid of microbes will have lower germination success and delayed germination phenology. Conversely, if partners co‐occur, we predict that interaction strength will increase with increasing stress (David et al., 2020), such that germination success and phenology are unaltered despite high‐stress (novel range or drought) conditions. To investigate the potential consequences of short‐term loss of the interactions among legumes and soil microbes, we ask whether the success and timing of germination are affected by (i) soil sterilization, (ii) foreign soils collected from elevations higher than the current distributions of these legumes, or (iii) limited soil moisture. Overall, we aim to address how disruptions in plant–soil microbe mutualisms may prevent range expansion and affect plant germination traits.

## 2 METHODS

### 2.1 Study system

This study was conducted using seeds and soils collected from The Rocky Mountain Biological Laboratory (RMBL; N 38° 52.2928′, W 106° 58.671′) located in the Maroon Bells‐Snowmass Wilderness area near Gothic, Colorado, USA. The RMBL area is characterized by vast, open subalpine meadows dominated by perennial wildflowers and patches of aspen‐fir forests. Subalpine plant communities, like those at RMBL, are especially sensitive to changes in climate due to short growing seasons and upward range limitations (Hülber et al., 2010; Parmesan, 2006). These subalpine plant communities therefore offer an excellent system to address questions on climate change effects on plant–soil microbe interactions In the last several decades at RMBL, snowpack has decreased, the date of spring snowmelt has shifted earlier (3.5 days earlier per decade from 1974–2012), and year‐to‐year variation in snowmelt date increased by 20% from 1974 to 2008 (CaraDonna et al., 2014; Lambert et al., 2010). Additionally, June precipitation has decreased significantly since the 1980s and July monsoon rains are delayed or nonexistent (data accessible at https://www.gothicwx.org/). Temperatures at RMBL have risen to date and are expected to continue to increase over the next century and total precipitation is expected to decrease (Overpeck & Udall, 2010). Decreased snowpack, earlier snowmelt, and decreased June precipitation are predicted to result in earlier, longer dry seasons prior to July monsoon rains (Clow, 2010; Kittel et al., 2015), which is likely to affect the species and their interactions in this system.

### 2.2 Selection of plant species

**FIGURE 1:**
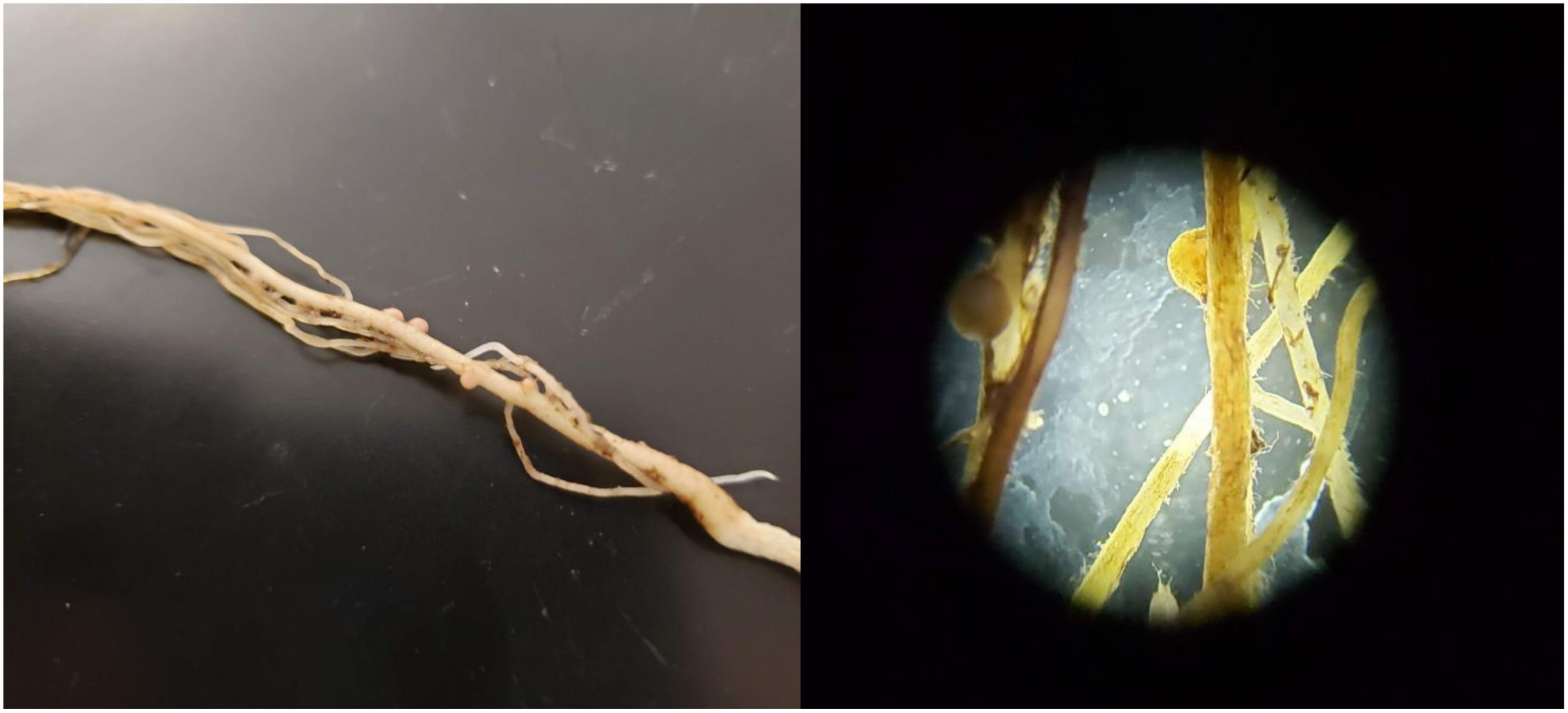
Root nodules found on plants grown in unsterilized, current range soils.

We studied the two native, nectar‐producing legume plant species present in the Maroon Bells‐Snowmass Wilderness area, *Lathyrus lanszwertii* var. *leucanthus* and *Vicia americana* (Fabaceae). Both are perennial vines that are common in the RMBL area and produce nutrient‐rich rewards that attract native pollinators. We verified that both of these species form root nodules and host N‐fixing bacteria in the field and in controlled common garden settings (Figure 1), and that they host arbuscular mycorrhizal fungi (AMF) and dark septate endophytes (DSE) in the field. These species are known to host AMF across their range and facultative DSE at higher elevations near RMBL (unpublished data, RMBL). However, after using previously established methods for alpine plants (Schmidt et al., 2008), staining and microscopy revealed neither fungal group on or in the roots of these legumes in our controlled common garden setting, likely because fungal spores can quickly decay in cool, wet soils if stored there for a month or more (Gottlieb, 1950; Varga et al., 2015), as our soils were. We note that, using the same staining methods, we have verified the presence of AMF in roots of other species grown from seeds collected at some of the same sites near RMBL, increasing our confidence that AMF were absent in our soils for this study. Along with AMF, DSE, and rhizobia, it is likely that various phylotypes of Acidobacteria, nitrifying taxa (e.g., *Nitrospira* spp. and *Thaumarchaeota* spp.), *Thelephora* (Agaricomycetes), *Hebeloma* (Agaricomycetes), *Archaeorhizomyces* (Archaeorhizomycetes), *Tetracladium* (Leotiomycetes), and other endophytes, such as fine root endophytes (FRE), were present in our soils, as these taxa are common in the soils around RMBL after snowmelt (Orchard et al., 2017; Sorensen et al., 2020).

*Vicia americana* is widely distributed across North America while *L. leucanthus* is located solely in mountainous regions of western North America. The ranges of *L. leucanthus* and *V. americana* in the RMBL region extend from ~2700 to 3500 m in elevation. Observational data suggest that the elevational ranges of both species (and their bumblebee pollinators) have expanded upward in the last 40 years, and leading edges are expected to continue to expand (Pyke, 1982; Pyke et al., 2012; Pyke et al., 2016). These observations come from systematic surveys conducted in the 1970s (Pyke, 1982), wherein the presence/absence of *L. leucanthus* and *V. americana* was noted along transects that span elevational gradients in the RMBL area, and the systematic resurveying in 2015–2018 of some of those same transects and others that span similar elevations (described herein). For example, whereas neither species was recorded as present at the highest‐elevation survey point (3394–3442 m) along the Washington Gulch transect in the 1970s (Pyke, 1982), both species were found there in 2015–2018. In the 1970s, the highest‐elevation survey point at which the focal legumes were documented was 3333–3393 m (Pyke, 1982).

### 2.3 Collection of soils and seeds

**FIGURE 2:**
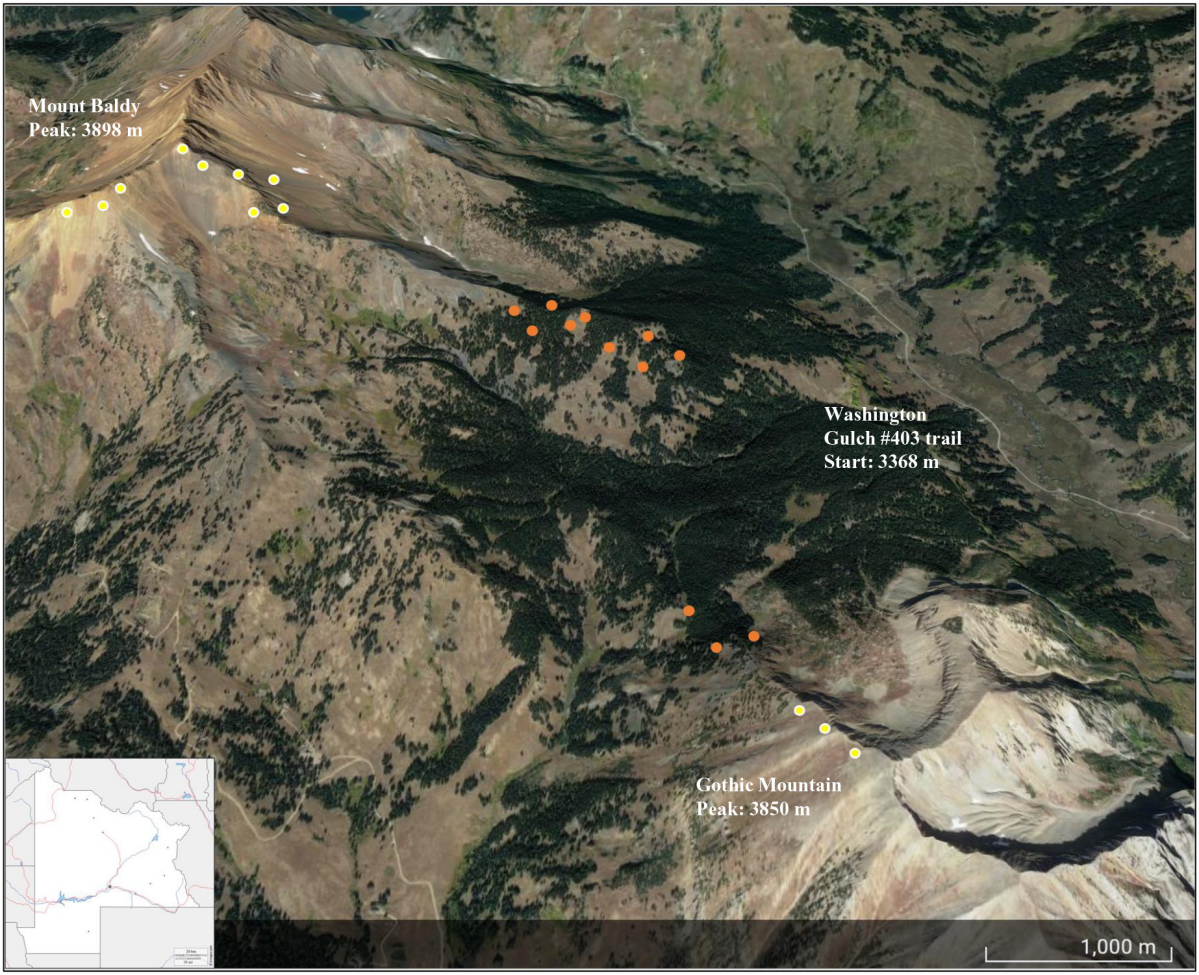
Soil collection sites at RMBL in Gunnison County, CO. Yellow dots are approximate areas where soil was collected beyond the current range of the legumes on mt. Baldy (upper left peak; 38.9926°N, 107.0462°W) and Gothic Mountain (lower right peak; 38.9564°N, 107.0106°W). Orange dots are approximate locations where soil was collected near *L. leucanthus* and *V. americana* patches.

We collected soils and seeds from multiple populations across gradients that span the current and expected future elevational ranges of our focal plant species at RMBL in summer 2017 and summer 2018 to understand how the loss of microbial partners, soil origin, and drought may affect subalpine legume germination traits. Soils and seeds were collected within 10 m of transects that traverse the Washington Gulch (403), Gothic Mountain, and Baldy Mountain trails (3200–3500 m in elevation; Figure 2). Within the current range of our focal legume species, soils were collected from within a 10 cm radius of the nearest legume to a depth of 15 cm, just past the rooting depth of *L. leucanthus* and *V. americana*, and where beneficial soil microbial species are likely to be at higher densities in the soil (Komatsu & Simms, 2020). A soil corer was centered over a focal legume and soils were exhumed from that core. To collect soils from elevations beyond the upper range limits of *L. leucanthus* and *V. americana* populations (>3500 m, just beyond treeline), we sampled at least three sites per transect after verifying that neither species occurred at those elevations, which ranged from 3500 to 3800 m. Similar to lower elevations, soils were collected from a 10 cm radius, 15 cm deep core. As no focal legumes were present at higher elevations, collection sites were chosen haphazardly; collections were made near plants such as *Lupinus argenteus*, *Castilleja* species, and various grasses and rushes. All soils were put on ice and transported back to the RMBL field station where they were stored at 4°C. Soils were kept on ice for a day during transportation to UC Riverside, then stored at 4°C. Soils from each elevational zone were homogenized each year to standardize nutritional content and soil texture. Seeds were haphazardly collected from mature fruits (i.e., pods) within 10 m of these same transects; seeds were collected from plants if pods were beginning to dehisce. One to six pods were collected per maternal plant, and each pod contained one to three seeds. Only unparasitized seeds were used in experiments. In total, 347 *L. leucanthus* and 1059 *V. americana* seeds were collected and used for these experiments. Seeds were cold stratified at 4°C for 45 days, the recommended time for these species (personal communication, Mike Bone, Denver Botanic Gardens). All seeds were weighed to the nearest 0.1 mg before sowing, except for the *V. americana* seeds sown in the drought experiment. The average seed mass was 15.7 ± 7.4 mg (mean ± SD) for *L. leucanthus* and 10.9 ± 8.7 mg for *V. americana*.

### 2.4 Experimental design and setup

To assess the effects of drought and soil origin, we designed two separate common garden studies (Table 1). We grew our focal legume species in sterile background soils inoculated with field‐collected soils (Collins, 2019; David et al., 2020). To control for abiotic differences across soil collection points, background soils were small‐batch sterilized (double autoclaved within 12 h at 121°C for 90 min, containers 0.5–1.5 L) and then were added to sterile pots (66 ml with drainage holes; Ray Leach Cone‐tainer, Stuewe & Sons, Tangent, Oregon, USA); background soils consisted of 57% sand, 43% peat moss, and various minerals. This relatively high ratio of inoculum to background soils was used in the likely case of low microbial biomass in soil samples. Although the majority of the soil in each pot was the same across treatments, there may have been slight differences in the abiotic properties of inoculants. We then added field‐collected soil inoculum to these sterile background soils; 85% of the total soil volume was made up of sterile background soil, while the other 15% of total soil volume was made up of field‐collected soil inoculum. Background and inoculum soils were thoroughly mixed. In addition to twice sterilizing pots and background soils, half of all field‐collected soils were twice autoclaved prior to use; in sterile soils, there can be no interactions between legumes and soil microbes, mimicking a complete loss of the possible mutualisms. These soils are referred to as sterile herein, but autoclaving does not ensure sterility; these soils are nearly sterile and contain very little, if any, microbial biomass.

**TABLE 1:** Experimental design showing the number of individually potted seeds planted in each soil type.

Soil type	Seed count: *L. leucanthus*	Seed count: *V. americana*
Unsterilized, current range	95	76
Unsterilized, beyond the current range	78	59
Sterilized, current range	96	71
Sterilized, beyond the current range	78	53
Unsterilized, well‐watered	n/a	200
Unsterilized, drought	n/a	200
Sterilized, well‐watered	n/a	200
Sterilized, drought	n/a	200

**FIGURE 3:**
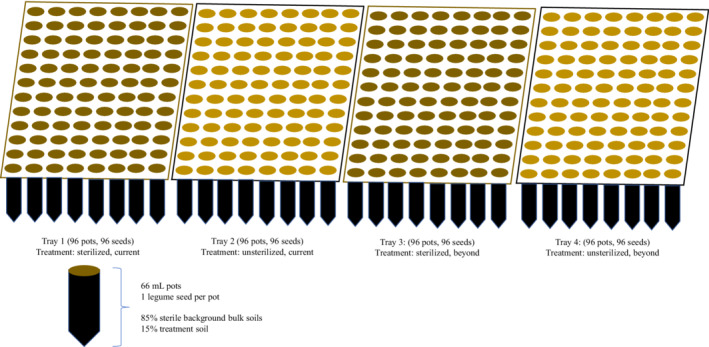
Common garden experimental design. Each tray contained a single treatment type to reduce movement of water and/or microbes between pots. There were 96 pots and 96 seeds per tray (one seed per pot). We show four example trays, but there were 15 total trays.

Wild‐collected *L. leucanthus* and *V. americana* seeds were surface sterilized for 20 min in a 10% bleach solution, briefly soaked in four subsequent sterile water baths, then allowed to dry (Collins, 2019; Oyebanji et al., 2009); seeds were sterilized to isolate the effects of the soil type and moisture treatments. Individual sterile, dry seeds were weighed, sown directly into separate soil‐filled pots at the same depth, then covered with ~10 g of treatment soil (one seed per pot). Each treatment soil was housed on a separate tray to reduce movement of microbes from pot to pot via watering or air circulation (Figure 3). Legumes were placed in a growth chamber (Conviron MTR30) at a temperature and photoperiod regime reflecting that of the early growing season (germination period of the focal species) at RMBL (day: 21°C, 50% relative humidity; night: 4.4°C, 20% relative humidity; 12:12 h light: dark cycle).

### 2.5 Legume germination traits in sterilized soils and soils from beyond the current elevational range: Implications for upward range expansion

We grew individuals of both legume species in separate factorial experiments that manipulated the presence of soil microbes (unsterilized vs. sterilized soil conditions) and elevational origin of the soil (within the current ranges vs. beyond the current ranges of the focal legumes) in soils collected from RMBL. Soil treatments comprised the following: unsterilized, current range soils (unmanipulated, microbially active, current range of the focal legumes; *L. leucanthus*: *n* = 95 seeds; *V. americana*: *n* = 76 seeds); unsterilized, beyond the current range soils (unmanipulated, microbially active, beyond the current range of the focal legumes; *L. leucanthus*: *n* = 78 seeds; *V. americana*: *n* = 59 seeds); sterilized, current range soils (double autoclaved, microbially sterile, current range of the focal legumes; *L. leucanthus*: *n* = 96 seeds; *V. americana*: *n* = 71 seeds); and sterilized, beyond the current range soils (double autoclaved, microbially sterile, beyond the current range of the focal legumes; *L. leucanthus*: *n* = 78 seeds; *V. americana*: *n* = 53 seeds; Table 1).

### 2.6 Legume germination traits in dry soil conditions: Implications for climate change‐induced soil drying

Separately, we grew *V. americana* in factorial experiments that manipulated the presence of microbes (unsterilized vs. sterilized conditions) and the soil moisture level (dry vs. well‐watered), where all soils were from within the current range and began at the same soil moisture VWC%. Half of all pots were placed into the well‐watered treatment while the other half were placed in the dry treatment. The well‐watered treatment was watered with sterile, twice autoclaved water every other day for 10 weeks while the dry treatment was watered every other day for 2 weeks then once every week for 8 weeks; approximately 3 ml of water was added to each pot at every watering. Soil treatments comprised the following: unsterilized, well‐watered soils (*n* = 200 seeds); sterilized, well‐watered soils (*n* = 200 seeds); unsterilized, dry soils (*n* = 200 seeds); and sterilized, dry soils (*n* = 200 seeds; Table 1).

### 2.7 Data collection

Seedling germination phenology, or the date of germinant emergence from the soil, was monitored every other day for 10 weeks. In total, we documented the timing of germination for 225 seeds (16% of the 1406 seeds sown germinated). Many alpine species, including our focal legumes, are highly clonal and are therefore expected to have a low rate of germination (Angevine, 1983; Callaghan et al., 1992; Eriksson, 1989). Indeed, germination success in both species tends to be low (4–11%) in natural conditions around RMBL and in laboratory settings, even when methods to crack the seed coats (e.g., sulfuric acid treatment) are used (personal communication, Mike Bone, Denver Botanic Gardens; unpublished data, N. E. Rafferty). Seeds that did not successfully germinate within 10 weeks were removed from pots and replanted in unsterilized soils with adequate water and monitored for 10 additional weeks; none of these seeds germinated after replanting.

### 2.8 Data analysis

To examine variation in germination success in soils that differed in soil sterility and elevational origin, we constructed generalized linear models (GLM) with binomial error. To investigate variation in germination latency in soils that differed in soil sterility and elevational origin, we used linear models (LM). To these models (with either germination success or latency as the response), we introduced species, seed mass, soil elevational origin, and soil sterility as predictors. The importance of seed mass for germination success or latency may depend on soil type, such that seed mass may be positively related to germination in sterilized soils but less important for seeds in unsterilized soils; we therefore also included the three‐way interaction between seed mass, soil origin, and soil sterility as a predictor. Because species was a significant predictor in all models, we fitted separate models for *L. leucanthus* and *V. americana*. We used the same approach to examine variation in germination success and latency in response to soil moisture, except we omitted species and seed mass as predictors because only *V. americana* was used in that experiment and the seeds were not weighed. We used likelihood ratio tests to compare the fit of nested models, starting with the full model and comparing the fit of reduced models, and we report the best‐fitting models. If a three‐way interaction was significant, then we retained all two‐way interactions and main effects in the model. Post hoc Tukey tests were used to test for significant pairwise differences between categorical soil treatments. All analyses were conducted in R version 4.0.2 (R Core Team, 2019).

## 3 RESULTS

### 3.1 Interactive effects of soil sterility, soil elevational origin or soil moisture treatment, and seed mass

**FIGURE 4:**
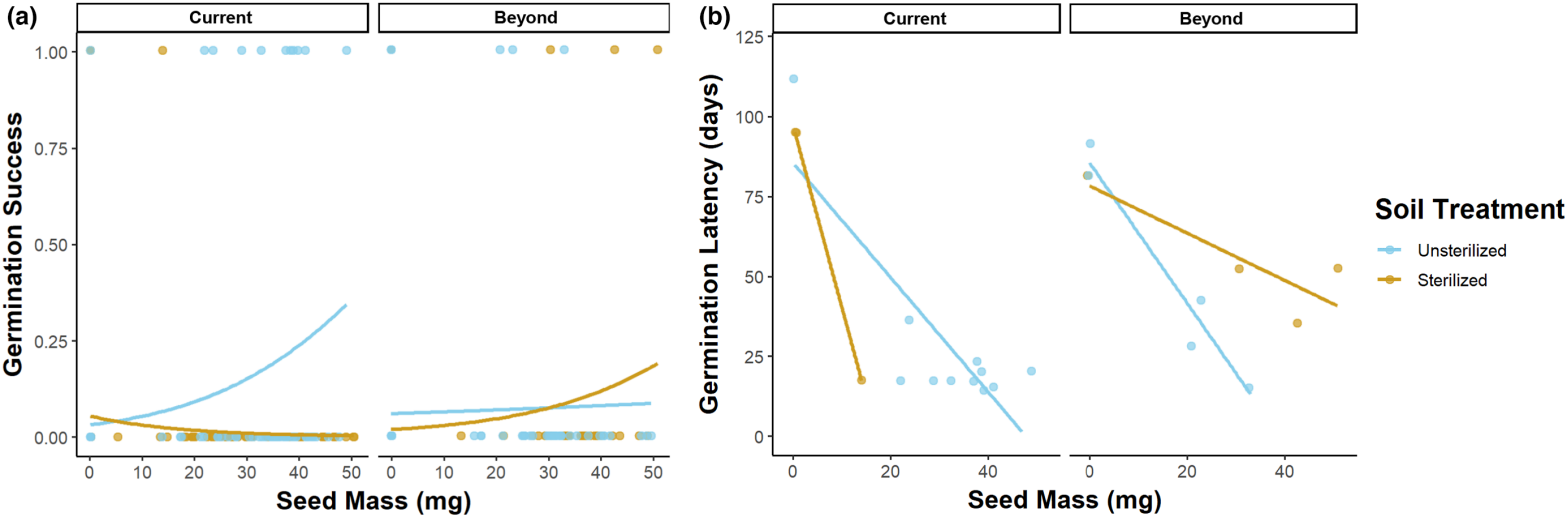
Germination success (a) and germination latency (b) of *L. leucanthus* depended on the interaction between seed mass, soil treatment (unsterilized vs. sterilized), and soil origin (current = within the elevational range; beyond = beyond the current elevational range). In current range soils, heavier seeds were more likely to germinate in unsterilized soils than in sterilized soils (a, current). Beyond the current elevational range, greater seed mass increased germination success in sterilized soils (a, beyond). Greater seed mass advanced germination to varying degrees, depending on soil type (b). Lines represent binomial (for germination success) or linear (for germination latency) fits.

We start by describing the best‐fitting models for each species, first for germination success and then for germination latency, before turning to specific contrasts of interest. For *L. leucanthus* germination success in the context of soil elevational origin, we detected a significant three‐way interaction between soil sterility, soil elevational origin, and seed mass, indicating that the effects of seed mass on germination success depended on soil type (GLM: −0.16 ± 0.07, *z*_346_ = −2.17, *p* < .03; Figure 4). For *V. americana* germination success relative to soil elevational origin, the best‐fitting model included only soil sterility, soil origin, and the interaction between the two as predictors. Thus, for this species, seed mass did not significantly affect germination success. For *V. americana* germination success in the context of soil moisture, the best‐fitting model included only the main effect of water treatment and no interactions.

For *L. leucanthus* germination latency relative to soil elevational origin, the best‐fitting model included a significant three‐way interaction between soil sterility, soil origin, and seed mass (LM: −5.33 ± 1.46, *t*_15_ = −3.65, *p* < .002; Figure 4). For *V. americana* germination latency pertaining to soil elevational origin, only soil sterility and seed mass were retained as predictors in the best‐fitting model. For *V. americana* germination latency in the context of soil moisture, no significant interactions were detected, and the best‐fitting model included only soil sterility and soil moisture treatment.

### 3.2 Legume germination traits in sterilized soils

**FIGURE 5:**
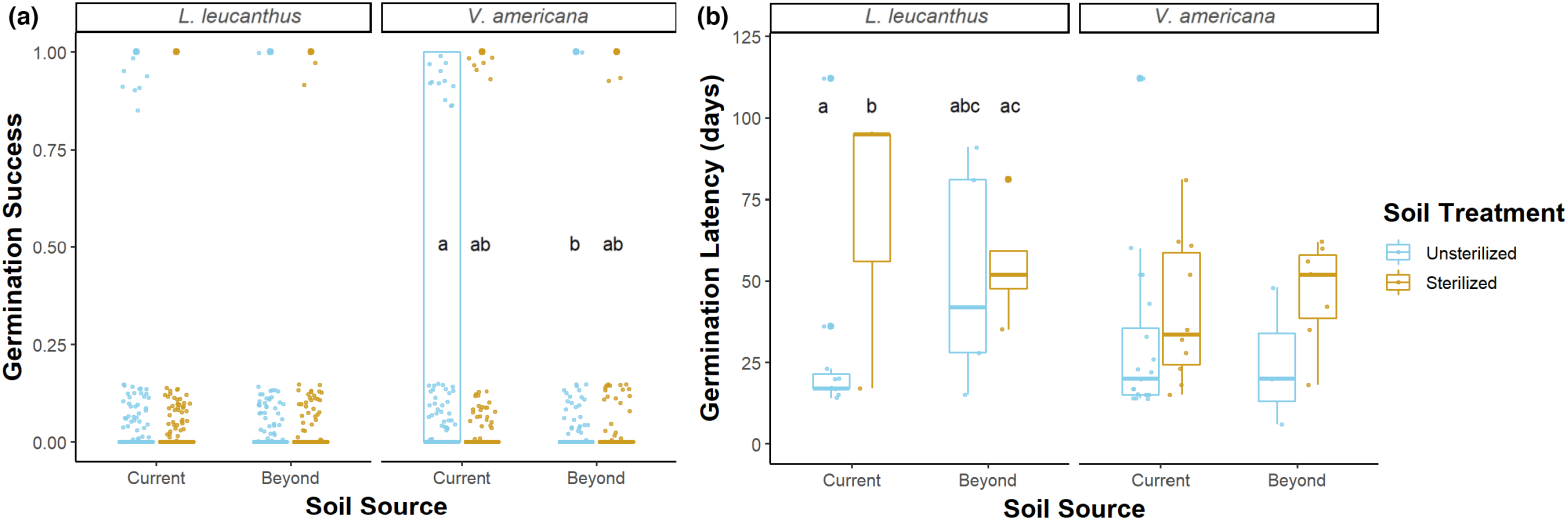
Germination success (a) and germination latency (b) for *L*. leucanthus and *V. americana* seeds by soil treatment (unsterilized vs. sterilized) and soil origin (current = within the elevational range; beyond = beyond the current elevational range). Microbial absence (sterilized soil) did not significantly alter *L. leucanthus* or *V. americana* germination success (a), but significantly delayed *L. leucanthus* germination within the current range (b; compare unsterilized, current vs. sterilized, current); this trend approached significance for *V. americana*. *Vicia americana* seeds planted in soils from beyond their elevational range had lower germination success than seeds planted in soils from within their current range (a; compare unsterilized, beyond vs. unsterilized, current). Points are jittered for clarity. Each bolded bar represents the mean germination value within the interquartile range.

There was no effect of soil sterilization on *L. leucanthus* or *V. americana* germination success. Germination success of *L. leucanthus* did not differ in sterilized vs. unsterilized soils from the current range (Tukey test: *p* < .16; Figure 5). However, in unsterilized, current range soils, heavier *L. leucanthus* seeds were more likely to germinate (Figure 4). Similarly, for *V. americana*, germination success did not differ in sterilized vs. unsterilized current range soils (Tukey test: *p* < .13; Figure 5).

For *L. leucanthus* in current range soils, the onset of germination in sterilized soils was delayed by about 2 weeks compared with germination in unsterilized soils (Tukey test: *p* < .03; Figure 5). In unsterilized, current range soils, *L. leucanthus* seeds germinated 28.0 ± 14.8 (mean ± SE) days after sowing, while in sterilized soils, seeds germinated in 69.0 ± 26.0 days. In unsterilized, current range soils, heavier seeds germinated more quickly (Figure 4). For *V. americana*, the delay in germination in sterilized vs. unsterilized current range soils approached significance (Tukey test: *p* < .06; Figure 5); seeds in unsterilized current range soils germinated in 30.2 ± 5.3 days vs. 40.0 ± 6.9 days in sterilized soils. Nodules were found on some of the plant roots of both species in the unsterilized but not in the sterilized treatments.

### 3.3 Legume germination traits in soils from beyond the current elevational range: Implications for upward range expansion

Germination success of *L. leucanthus* did not differ in unsterilized soils from within vs. beyond the current elevational range (Tukey test: *p* < .71; Figure 5). There was no difference in germination success in sterilized vs. unsterilized soils from beyond the range (Tukey test: *p* < .98). However, in sterilized soils from beyond the range, heavier *L. leucanthus* seeds had a higher probability of germination (Figure 4). For *V. americana*, germination success was significantly lower in unsterilized soils from beyond the current elevational range compared to that within the range (Tukey test: *p* < .01; Figure 5). There was no significant difference in germination success between sterilized vs. unsterilized soils collected from beyond the current elevational range (Tukey test: *p* < .71).

Seeds of *L. leucanthus* and *V. americana* sown in unsterilized soils from within vs. beyond their current elevational range showed no significant difference in germination timing (*L. leucanthus* Tukey test: *p* < .73; *V. americana* Tukey test: *p* < .95; Figure 5). In unsterilized, beyond the current range soils, heavier *L. leucanthus* seeds germinated more quickly (Figure 4). Notably, *L. leucanthus* seeds germinated more quickly in sterilized beyond the current elevational range soils than in sterilized current range soils (Tukey test: *p* < .01), and this was not the case for *V. americana* (Tukey test: *p* < .94). Regardless of soil origin, heavier *V. americana* seeds germinated more quickly than lighter seeds (LM: −1.83 ± 0.32, *t*_15_ = −5.75, *p* < .00001).

### 3.4 Legume germination traits in dry soil conditions: Implications for climate change‐induced soil drying

**FIGURE 6:**
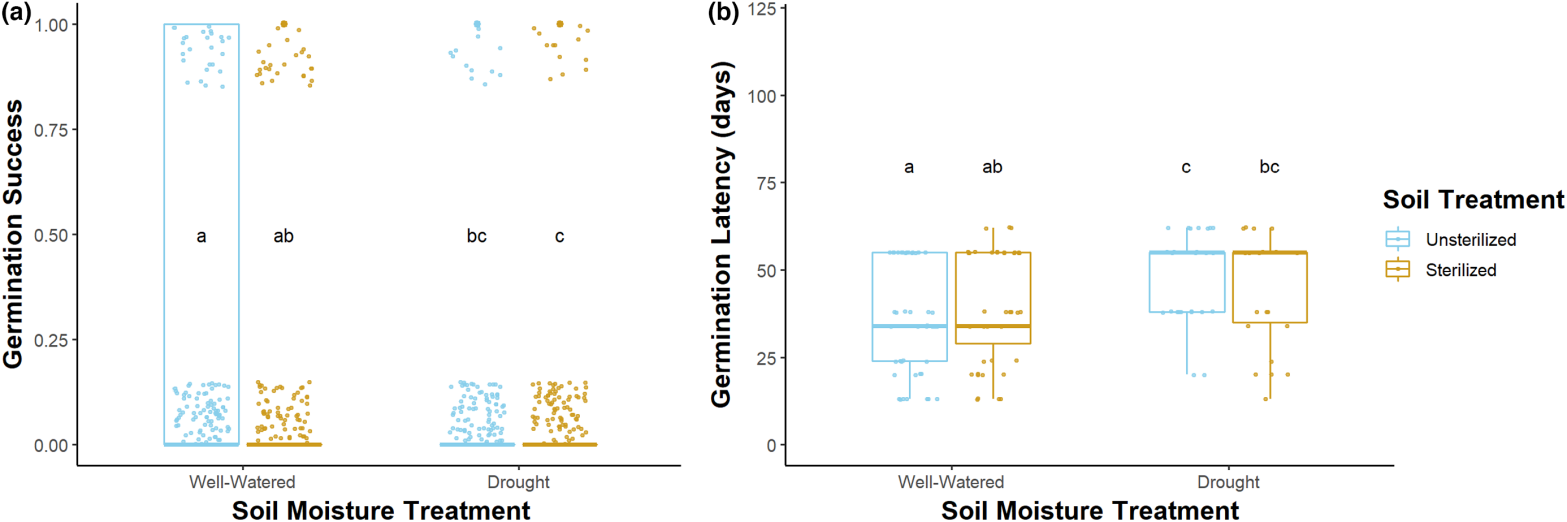
Germination success (a) and germination latency (b) for *V. americana* seeds by soil treatment (unsterilized vs. sterilized) and soil moisture treatment (well‐watered vs. drought). Germination success was lower in unsterilized, dry soils than in unsterilized, well‐watered soils (a). In dry soils, unsterilized or sterilized, seeds germinated later than those in unsterilized, well‐watered soils (b). Germination success and timing did not differ significantly between seeds planted in dry conditions (unsterilized, drought vs. sterilized, drought) or well‐watered conditions (unsterilized, well‐watered vs. sterilized, well‐watered). Points are jittered for clarity. Each bolded bar represents the mean germination value within the interquartile range.

*Vicia americana* seeds in unsterilized, dry soils had lower germination success than those in unsterilized, well‐watered soils (Tukey test: *p* < .03; Figure 6). Germination success was 26 ± 2% in unsterilized, well‐watered soils, higher than the 15 ± 2% seen in unsterilized, dry soils. Similarly, there was greater germination success in sterilized, well‐watered conditions vs. sterilized, drought conditions (Tukey test: *p* < .01). Seeds in unsterilized, dry conditions also experienced delayed germination compared with those in unsterilized, well‐watered soils (Tukey test: *p* < .0001; Figure 6). In unsterilized soils under dry conditions, seeds germinated in 50.2 ± 1.8 days, while in unsterilized soils under well‐watered conditions, seeds germinated in 35.2 ± 1.9 days. Lastly, *V. americana* seeds planted in unsterilized, well‐watered soils germinated more quickly and readily than those in sterile, dry conditions (germination latency Tukey test: *p* < .01; germination success Tukey test: *p* < .001; Figure 6).

## 4 DISCUSSION

Short‐term weakening or loss of beneficial partnerships is becoming increasingly common as our climate rapidly changes, and repeated instances of loss can lead to a mutualism breakdown (Kiers et al., 2010; Werner et al., 2018). The loss of a mutualism can alter the distribution, functional traits, and survival of both partners. In this study, we found that a forced loss of interaction between legumes and root mutualists can have consequences for plant traits, where legume germination timing, but not success, was negatively affected by sterilized, microbe‐free soils. Specifically, our results demonstrate that the onset of germination of *L. leucanthus* in sterilized soils depleted of microbes was delayed by about 2 weeks (Figure 4). However, germination probability was low for *L. leucanthus*, with only a few seeds germinating in sterile conditions, suggesting further study is warranted. Similarly, delays in germination in *V. americana* approached significance. These results suggest that some legume seeds and seedlings may benefit from interacting with plant growth‐promoting soil microbes shortly after germinating and possibly even before germination via germination stimulation. Germination timing is a particularly important trait, as it affects a plant's competitive interactions and hardiness to frost and drought, and individuals that survive this fragile life stage are more likely to reach reproductive stages. The observed stimulation of germination by soil microbial mutualists is well known in systems such as the orchid‐fungal mutualism (Arditti, 1967; Dressler, 1981), in certain legume‐rhizobia mutualisms (Miransari & Smith, 2009), and in plant interactions with *Pseudomonas fluorescens* (Moeinzadeh et al., 2010), but this is the first known evidence of this phenomenon occurring in the legumes of this subalpine system. Further research on the timing of this stimulation, by isolating initial shoot growth from seed splitting at germination, will allow us to better understand the effects of these seed–microbe interactions (Walsh et al., 2021). This work contributes to the growing number of findings that the community of soil microorganisms around a seed influences germination timing, which may affect plant fitness (Mordecai, 2012; Lamichhane et al., 2018; Das et al., 2019; Eldridge et al., 2021).

As climatic patterns shape the natural distributions of species, changing climate conditions strongly influence species' ranges (Becker‐Scarpitta et al., 2019; Chen et al., 2011), typically promoting range expansion toward higher latitudes and elevations and range contractions away from lower latitudes and elevations (Davis & Shaw, 2001; Lenoir & Svenning, 2015; Parmesan, 2006). Because the presence of mutualists can serve to expand the range of a partner by ameliorating abiotic stressors in novel environments (Afkhami et al., 2014; Halvorson et al., 1991; Harrison et al., 2018; Hayward et al., 2015; Stachowicz, 2001) and the absence of a mutualist can constrict the range of a partner (Harrower & Gilbert, 2018; Nuñez et al., 2009; Simonsen et al., 2017), these mutualist‐hosting legumes may not be able to expand their elevational ranges upward if compatible soil microbes are not present beyond the current range. This study demonstrates that the leading range edges of *L. leucanthus* may not be restricted to 3500 m as active, compatible microbial species may be present at higher elevations; germination timing and success of this species in higher‐elevation soils mirrored that in current range soils. Abiotic soil properties at higher elevations may also be conducive to earlier germination of *L. leucanthus*, as seed germinated more quickly in sterilized soils from beyond the range than in sterilized soils from within the range, although very few seeds of this species germinated in sterile conditions. In contrast, *V. americana* germination success was over five times lower in novel, beyond the range soils, indicating that beneficial microbe strains specific to *V. americana* plants may be absent or at low abundances at higher elevations, leading to reduced germination success. Although sequencing and quantification of both within‐ and beyond the range soils are needed to confirm the presence and abundance of active microbes, the comparable germination timing and success in *L. leucanthus* in novel vs. current elevational range soils suggests that compatible *L. leucanthus*‐specific soil microbes may facilitate the leading range expansion of this legume.

Germination‐triggering soil wetting events are becoming less frequent and less intense in many areas due to climate change (Saatkamp et al., 2019). One of the predictions of the stress gradient hypothesis is that mutualistic interactions increase in strength with increasing stress (Callaway et al., 2002; David et al., 2020). Legumes grown in stressful conditions may not exhibit reduced germination success and initial survival relative to those in less stressful conditions as long as microbial mutualists can buffer the abiotic stress by stimulating germination, provisioning N, P, and water, and reducing root parasitism, thereby increasing plant performance (Figueiredo et al., 2008; Jemo et al., 2017; Marinković et al., 2019; Pawar et al., 2014; Tankari et al., 2019). Conversely, stressful, dry soils promote microbial dormancy, preventing beneficial microbes from stimulating germination or interacting with the seed or plant (de Vries et al., 2018). In this controlled common garden study, legume seeds subjected to dry conditions were less likely to germinate and had significantly delayed germination compared with those in well‐watered soils, likely due to stressful abiotic conditions for both the seed and the microbes. This reduced germination stimulation may reflect a drought‐induced loss of the mutualism. If a plant germinates later than the optimal time, plant–plant competition will be greater and the ability to acquire limited resources such as water, light, P, and N will be reduced (Leverett, 2017; Lloret et al., 1999). Downstream phenological patterns, such as flowering time, could also be delayed, affecting pollinator visitation rates and reproductive output (Rafferty & Ives, 2012).

Although microbes compatible with *L. leucanthus* may be present at high elevations, abiotic conditions at these elevations are relatively harsh; low soil moisture, high UV exposure, and high winds, among other factors common above the treeline, could limit focal legume establishment beyond 3500 m (Normand et al., 2009). In addition to this, mammalian seed and seedling herbivores may limit seedling establishment in novel areas (Bueno de Mesquita et al., 2020; Lynn et al., 2021). In this study, stressful, dry conditions led to decreased percent germination and delayed germination; drier alpine conditions may not allow this mutualism to establish or persist and may hinder a continued upward range expansion. Interestingly, *Lupinus argenteus*, a co‐occurring rhizobia‐ and AMF‐hosting legume, occupies higher elevations than *L. leucanthus* or *V. americana*. The PGPR which interact with *L. argenteus* may stimulate the germination of other legume species (Hirsch et al., 2001), although sequencing of both within‐ and beyond the range soils is needed to confirm the observational evidence of soil microbes occurrence in both ranges. Another avenue to be explored is that of the seed microbiome (Nelson, 2018), specifically the epiphytic microbial community for horizontally transmitted mutualists such as rhizobia and other PGPR bacteria. The seed coats of both focal legume species are not smooth; if seeds fall to the ground and accumulate mutualists before dispersal, an upward range expansion may be more likely to occur because the partners would co‐occur spatially, although joint dispersal of legumes and rhizobia and other microbes has seldom been studied (Porter et al., 2018; Wendlandt et al., 2021), and joint dispersal does not always imply interaction (Wornik & Grube, 2010). Sequencing of soils and dispersed seeds would be useful to test this possibility.

In addition to soil microbe‐mediated germination stimulation, seed traits, such as seed mass, are important components of germination success and timing (Lord et al., 1995; Thompson, 2008; Venable et al., 1998). It is thought that heavier seeds are an adaptation for overcoming stressful conditions, such as drought, during seedling establishment (Wullf, 1986), as larger seeds increase seedling persistence via greater internal resource provisioning (Harrison & LaForgia, 2019; Lebrija‐Trejos et al., 2016; Leishman & Westoby, 1994). The transition from seed to seedling can be a defining period in the life history of a plant (Larson et al., 2015; Muscarella et al., 2013), and here, we found that heavier *L. leucanthus* seeds germinated more quickly and tended to have greater germination success than lighter seeds. This trend was especially pronounced when seeds were planted in the most stressful conditions, sterile soils collected from beyond the current range of *L. leucanthus* (Figure 4). For *V. americana*, germination success was not affected by seed mass, but heavier seeds germinated earlier regardless of soil origin. In the absence of epiphytic microbes, other factors such as seed mass and abiotic components of the seed environment (e.g., soil moisture and available nutrients) become more important (Lamichhane et al., 2018). In these scenarios, heavier seeds are predicted to be more vigorous, and thus germinate at a higher and faster rate.

A drawback to using a sterile soil treatment is that sterilization removes not only plant growth‐promoting soil mutualists but also all other potential soil microbes. Based on field observations near the study area, *L. leucanthus* and *V. americana* interact not only with rhizobia but also with AMF and DSE (unpublished data, RMBL). Through staining and microscopy, neither of these fungal symbionts were found on or in any of the plant roots in this study, but nodules were found on some of the plants in unsterilized, current elevation soils. As simultaneous infection by multiple belowground mutualists can additively benefit the plant (Afkhami & Stinchcombe, 2016), future work that assesses the effects of native rhizobial, AMF, DSE, FRE, and other PGPR bacterial infection on legume functional traits would be valuable.

Symbiotic rhizobia in extreme environments have lost the genes responsible for the initiation and maintenance of their mutualism with legumes due to natural selection; the maintenance of this nonessential portion of the genome is costly to the bacteria in harsh environments (Denison & Kiers, 2004; Sachs et al., 2011; Sullivan et al., 1996; Sullivan & Ronson, 1998). The loss of this segment of DNA ultimately causes a complete breakdown of the mutualism, only likely after numerous short‐term losses of the mutualism. A climate change‐induced breakdown in the mutualism between legumes and rhizobia will have significant effects on legume germination, phenology, and N‐acquisition, which could affect higher‐order mutualists, such as pollinators (Keeler et al., 2021), and plant community structure (Suttle et al., 2007). Just as floral traits such as nectar quality can be directly related to soil nutrient availability (Burkle & Irwin, 2009; Mevi‐Schutz & Erhardt, 2005), short‐ or long‐term loss of the interaction between plants and soil microbial species due to mutualism loss or breakdown will indirectly affect floral traits by altering host plant nutrient acquisition (Ballhorn et al., 2013; Gwata et al., 2003; Megueni et al., 2006; Namvar & Sharifi, 2011), which could cascade to affect pollinator behavior and legume reproductive success (Keeler et al., 2021). The long‐term fitness consequences of this particular mutualism loss are generally unknown (Berg et al., 2010; Kiers et al., 2010), although slower growth and lower quality floral rewards in these pollinator‐dependent, pollen‐limited plants (Xingwen, 2021) may further decrease reproductive success and thus recruitment in a warming, drying climate.

In this controlled common garden study, we found evidence that active plant germination‐promoting microbial species enabled legume germination and advanced germination timing. Soil sterilization (and consequent microbial absence) and dry soils caused germination to be delayed by 2–5 weeks as a result of interaction loss between legumes and germination‐promoting soil microbes. Additionally, we documented the presence of beneficial soil microbes beyond the current elevational range of one of our focal legume species which may allow for expansion of the leading range edge of *L. leucanthus* but not *V. americana*, suggesting *V. americana* may require more specialized interactions with soil microbial species. As soils dry and changing climatic conditions reshape legume upper range edges, beneficial soil microbial species may become inactive or absent, altering legume germination timing and success and ultimately affecting legume demographies and interactions with other mutualists.

## AUTHOR CONTRIBUTIONS

**Andrea Marie Keeler:** Conceptualization (lead); data curation (lead); formal analysis (equal); funding acquisition (equal); investigation (equal); methodology (lead); project administration (equal); resources (equal); validation (equal); visualization (equal); writing – original draft (lead); writing – review and editing (equal). **Nicole E Rafferty:** Conceptualization (supporting); data curation (supporting); formal analysis (equal); funding acquisition (equal); investigation (equal); methodology (supporting); project administration (equal); resources (equal); supervision (lead); validation (equal); visualization (equal); writing – original draft (supporting); writing – review and editing (equal).

## Data Availability

Data Accessibility Complete germination success and phenology data are available on Dryad: https://doi.org/10.6086/D16380.
